# Human Cognitive Architecture as an Intelligent Natural Information Processing System

**DOI:** 10.3390/bs15030332

**Published:** 2025-03-07

**Authors:** Slava Kalyuga

**Affiliations:** School of Education, University of New South Wales, Sydney 2052, Australia; s.kalyuga@unsw.edu.au

**Keywords:** human cognitive architecture, intelligent natural information processing system, intelligence

## Abstract

Human cognitive architecture represents an intelligent natural information processing system that is described by six operational principles: *information store, randomness as genesis, borrowing and reorganizing, narrow limits of change, environmental organizing and linking, and explicit intention to learn principles*. The last principle, added recently, is critical, as it distinguishes this system from other, lower-level natural information-processing systems and is responsible for controlled information processing and explicit learning as opposed to implicit cognitive mechanisms of lower-level systems. The paper provides a theoretical overview of the updated model of intelligent natural information processing systems. In this model, the explicit intention to learn principle affects the operation of other principles and is directly related to intelligence as an emergent characteristic of such systems. Human intelligence and explicit, intentional learning (and motivation that is required for such learning to happen) caused the emergence of human culture on a distinct, grand scale in the process of transition from biological to cultural evolution. The paper concludes with some important educational implications emanating from the new model focusing on strengthening human intelligence.

## 1. Introduction

Human cognitive architecture could be classified as an intelligent natural information processing system that is described by six operational principles: *information store, randomness as genesis, borrowing and reorganizing, narrow limits of change, environmental organizing and linking, and explicit intention to learn principles* (see Kalyuga, 2023, 2025, for a recently suggested extension to the original five principles of natural information processing systems proposed by Sweller, 2003, 2022, based on an analogy with biological evolution by natural selection). In this context, principles are considered fundamental theoretical assumptions about the operation of a system.

The *explicit intention to learn* principle is believed to be essential to describe a system at the level of human cognition—the intelligent natural information processing system–in contrast to other, lower-level natural information processing systems that are described by the first five principles only. This principle is directly associated with intelligence (i.e., the ability to learn to handle novel situations across domains) as an emergent feature of natural information processing systems in the process of transit from biological to cultural evolution stages. It may also drive the transition to a technological phase of cultural evolution—the transition that humanity is about to start with the development of truly intelligent artificial information processing systems (strong AI).

This paper analyses the interactions of the explicit intention to learn principle with other principles of natural information processing systems and provides a theoretical overview of the suggested updated model of human cognitive architecture as an intelligent natural information processing system. It also draws some potential educational implications of these interactions that have not been considered previously in sufficient (if any) details in research on educational consequences of major characteristics of human cognitive architecture (for example, in cognitive load theory). The particular focus is on the evolutionary significant need to keep enhancing human natural intelligence.

## 2. Human Cognition as an Intelligent Natural Information Processing System

An evolutionary perspective on human cognitive architecture was originally proposed in cognitive load theory (a branch of instructional psychology) to provide an explanatory framework for major features of human cognitive architecture that affect learning and teaching processes (Sweller, 2003, 2022; Sweller et al., 2011). This perspective was framed as a set of five principles presumably common to all natural information processing systems, such as the human cognitive system, as well as other biological systems, including biological evolution by natural selection as a prime example of a natural information processing system. In fact, the information processing analogies between human cognition and biological evolution triggered the original formulation of the set of five principles: *information store, borrowing and reorganizing, randomness as genesis, narrow limits of change*, and *environmental organizing and linking* principles (Sweller & Sweller, 2006).

While these five principles describe well the operation of biological systems (including evolution by natural selection as a biological system) other than humans, they apparently fail to explain all aspects of human cognition, particularly the processes associated with controlled, conscious information processing. Humans can override implicit, evolved, automatically executed heuristics in favor of effortful, conscious reasoning and problem-solving. The addition of another principle—*the explicit intention to learn principle*—was suggested as a possible way to resolve this issue and describe higher-level information processing systems, such as human cognition, which were defined as intelligent natural information processing systems (Kalyuga, 2023, 2025). According to this view, the most important factor that distinguishes human cognition from the rest of the biological-level natural information processing systems is the emergence of intentionally controlled explicit mechanisms capable of overriding implicit, evolved responses. These processes take place in the form of conscious reasoning in human working memory, and they allow humans to effectively deal with novel environmental situations.

### 2.1. Explicit Intention to Learn Principle as a Determinant of Evolutionary Domains of Operation of Other Principles

Two evolutionary domains of operation of natural information processing systems refer to Geary’s (2005, 2007, 2008) separation of two major categories of knowledge and abilities based on their evolutionary origins. Biologically primary knowledge and abilities represent implicit intuitive knowledge, found in a species evolved to be genetically predisposed to acquire this knowledge rapidly and non-intentionally just by functioning in natural environments conducive to such abilities. For example, the abilities to listen and speak a basic native language are learned naturally while communicating within corresponding social groups. In contrast, biologically secondary knowledge is learned slowly and effortfully through conscious, controlled processing in working memory (e.g., writing and reading skills, or scientific knowledge).

**Figure 1:**
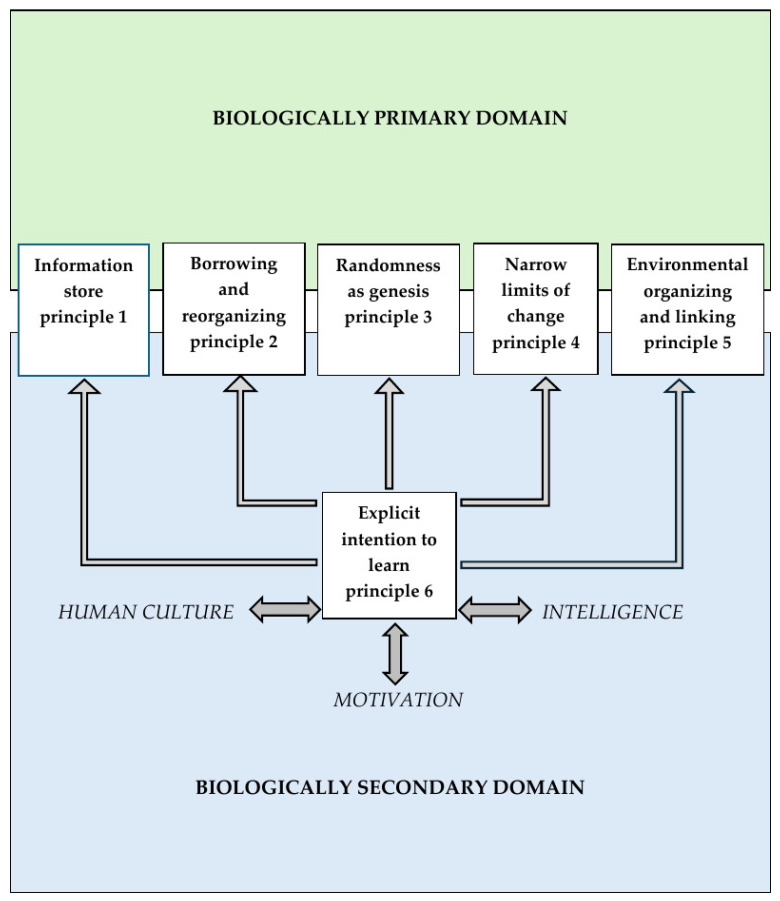
Graphical depiction of human cognitive architecture as an intelligent natural information processing system (including some key relations between the guiding principles, biologically primary and secondary knowledge domains, motivation, intelligence, and human culture).

The distinction between biologically primary and secondary domains has been compared to the distinction between intuitive (Type 1) thinking and analytical (Type 2) thinking in dual-process theories of reasoning (e.g., Barrouillet, 2011; Evans, 2008; Evans & Stanovich, 2013). Both primary domain processes and intuitive, Type 1 thinking are implicit, unconscious, and related to evolutionary rationality. On the other hand, both biologically secondary processes and analytical, Type 2 thinking are explicit, conscious, related to individual rationality, and dependent on working memory operation (Lespiau & Tricot, 2022a, 2022b). Whether the original five principles of natural information processing systems operate in biologically primary or secondary domains is likely to depend on the involvement of the explicit intention to learn principle (Figure 1).

#### 2.1.1. Information Processing in Biologically Primary Domains

If the five original principles act on their own, without the involvement of the explicit intention to learn principle, they operate in the domain of biologically primary, implicit knowledge, whether it is a human cognitive system (upper part in Figure 1) or other biological, lower-level information-processing systems. These five principles cover the major aspects of biologically primary information processing in the natural world, such as the storing of such information (principle 1), the acquisition of this information (principles 2 and 3), the processing of such information (principle 4), and the usage of the acquired information (principle 5).

*The information store principle* assumes that behavioral environmental responses of natural information-processing systems operating in biologically primary domains are controlled by the store of implicit, biologically primary information patterns (e.g., genome in the biological evolution system, implicit memory structures in human or non-human primates’ cognitive architectures).

*The borrowing and reorganizing principle* suggests that most of the biologically primary information patterns in the information store are borrowed directly from other sources (if available) and implicitly reorganized to fit the system (if necessary). For example, in the biological evolution system, genetic information in the genome of biological organisms is borrowed through sexual reproduction and reorganized to be incorporated into the genome; in human or non-human primates’ cognition, individuals borrow biologically primary knowledge and abilities (e.g., basic spoken language or other means of communication, social skills) from other individuals by observing and imitating their corresponding behavioral patterns. For instance, chimpanzees are capable of imitative learning even after a time delay (which suggests that they build some form of memory in the information store) (Bjorklund & Bering, 2003).

*The randomness as genesis principle* indicates that principally new biologically primary information (not available through borrowing) is acquired by random generate-and-test processes (e.g., random mutations in the biological evolution system, random trial-and-error or similar evolved implicit problem-solving heuristics in human or non-human primate cognition).

*The narrow limits of change principle* suggests that in a novel environment, the system invokes a mechanism that restricts the range of possible modifications to the already existing information patterns to avoid significant interruptions to their functionality. As an example, only a limited range of random mutations at a time is allowed in the biological evolution system which is determined by the epigenetic system. Similarly, short-term memory storage capacities of humans and non-human animals are also restricted in duration and capacity. For instance, non-human animals, including the great apes as our closest animal relatives, are not able to form lasting memories for arbitrary stimuli, which makes it difficult for them to learn even short stimulus sequences (Lind et al., 2023). It is believed that the great apes have a short-term memory span of 2 ± 1 (in contrast to 7 ± 2 in humans) (Read et al., 2022). Apparently, such low capacity is insufficient for controlled conscious reasoning abilities (like those provided by human working memory), but still effective in restricting the range of modifications to implicit primary knowledge structures.

*The environmental organizing and linking principle* suggests that if the system operates in a familiar environment, the restrictions on possible changes to the information store are significantly lifted. For example, in the biological evolution system, the epigenetic system switches appropriate genes on or off depending on the specific environment, thus affecting modifications in the genome and leading to different phenotypes; in human and non-human primates’ cognitive architectures, the relevant implicit biologically primary knowledge structures available in memory stores could lift the above memory limitations. This principle could be related to the concept of culture as a repository of nongenetic information that has accumulated over generations. While distinctly human culture (grand-scale cumulative culture) is based on biologically secondary information and will be referred to in the next subsection, there are possibly two lower-level, rudimentary forms of culture that are based on biologically primary information, such as (1) non-cumulative culture in non-human animals related to local sets of traditions and (2) evolved cumulative culture related to folk knowledge, traditions, and tools in humans (with some limited presence observed also in the great apes) (see Kalyuga, 2025, for a brief review).

#### 2.1.2. Information Processing in Biologically Secondary Domains

The explicit intention to learn principle was added to the previously formulated five principles of operation of natural information processing systems with the intent to account for the emergence of controlled, explicit processes hypothetically representing a qualitatively new evolutionary level of such systems (Kalyuga, 2023). If the explicit intention to learn principle is involved in the operation of a natural information-processing system, then, together with the original five principles, they operate in the domain of biologically secondary, explicit knowledge. Such a system (e.g., human cognitive architecture) presumably represents a higher-level information-processing system denoted as an intelligent natural information-processing system. Analyses of relations between psychological and evolutionary characteristics of intelligence and attributes of the explicit intention to learn principle supported the classification of such a six-principle, higher-level natural information-processing system as an intelligent natural information-processing system (Kalyuga, 2025). This inherent link between the explicit intention to learn principle and intelligence as the ability to generate new secondary knowledge is indicated in Figure 1.

Like in the case of biologically primary domains, the now six principles cover the major aspects of biologically secondary information processing in human cognitive architecture, such as the storing of secondary information (principle 1), the acquisition of such information (principles 2 and 3), the processing of biologically secondary information (principles 4 and 6), and the usage of the acquired secondary information (principle 5).

*The information store principle* assumes that the behavior of the system in complex natural, technological, and social environments is guided by the store of acquired biologically secondary information patterns (organized knowledge structures) accumulated over generations which constitutes a unique human cumulative culture providing motivational, cognitive and perceptual means for dealing with external environments (Henrich & Muthukrishna, 2021).

*The borrowing and reorganizing principle* suggests that the system acquires most biologically secondary information structures in a controlled, effortful way directly from external sources—other people, written and spoken texts, visuals, etc.— then this information is consciously reorganized and embedded into existing knowledge structures (as the process of cultural knowledge transfer between the generations).

*The randomness as genesis principle* indicates that principally new biologically secondary information (which is not available through borrowing) is acquired by search processes by explicitly, consciously applying heuristics-based general problem-solving methods such as means-ends analysis. A critical factor of problem-solving abilities in humans (as well as to a significant degree in non-human primates) is associated with inhibitory skills—the ability to inhibit previously used behavioral strategies mostly based on implicit (biologically primary) information store structures and flexibly modify behavioral responses in changing, novel conditions (Amici et al., 2008, 2018). Comparison studies indicated that the inhibitory skills of the great apes are roughly equivalent to the skills of human children aged around 5 years old (Vlamings et al., 2010). In humans, these abilities continue to develop further, resulting in much more advanced problem-solving and multitasking abilities (Tomasello & Herrmann, 2015). It is possible that the combination of both enhanced human inhibition abilities and working memory capacity supported the emergence of the ability to generate new secondary knowledge as the key defining factor of intelligence.

*The narrow limits of change principle* suggests that when the system operates in a new environment, the limited capacity and duration of working memory provide the mechanism that limits the scale of modifications to the existing knowledge structures in the information store (long-term memory). In human cognition as an intelligent natural information processing system, working memory with its capacity and duration limitations has two functions. On the one hand, according to the narrow limits of change principle, it prevents potentially detrimental significant modifications to the information store’s biologically secondary knowledge structures. On the other hand, working memory provides the above-mentioned inhibitory abilities and controlled, conscious information processing related to the explicit intention to learn principle.

*The explicit intention to learn principle* stipulates the capacity of the system to suppress potentially ineffective responses based on biologically primary information patterns in the information store and engage consciously controlled, explicit information processing in working memory to effectively deal with novel situations. This principle assumably affects the operation of each of the original five principles making them applicable to the biologically secondary domains and controlled processes leading to biologically secondary knowledge. The information processing mechanisms associated with the explicit intention to learn principle operate through conscious, effortful processing in working memory. In contrast to information processing in the primary domains in which humans or non-human animals are naturally motivated to acquire implicit, evolved patterns of information, humans need to be explicitly motivated to acquire biologically secondary knowledge by engaging in intentional, effortful, and goal-driven activities supported by working memory processes. There is an inherent connection between cognitive processes in the biologically secondary domain guided by the explicit intention to learn principle and motivation (as indicated in Figure 1).

*The environmental organizing and linking principle* suggests that there are no restrictions on changes to the biologically secondary knowledge structures in the information store when the system operates in familiar environments. In human cognitive architecture as an intelligent natural information processing system, the relevant knowledge structures available in long-term memory significantly reduce (if not eliminate) working memory limitations due to chunking mechanisms–by encapsulating many information elements into larger units. This is essentially the role of cumulative grand-scale human culture in providing efficient means for dealing with external environments based on explicit, biologically secondary abilities and symbolic representations.

## 3. Educational Implications of the Updated Model of Human Cognitive Architecture

Educational implications of the original five principles of natural information processing systems applied to human cognition have been well investigated within the framework of cognitive load theory as an instructional theory based on our knowledge of human cognitive architecture (e.g., Sweller, 2012, 2022; Sweller et al., 2011). Cognitive load theory generated many empirically supported instructional effects associated with the five original principles applied to the domains of biologically secondary knowledge. Since biologically secondary domains always require explicit, controlled information processing mechanisms, from the updated framework’s viewpoint, those applications could be considered as performed in a somewhat short-cut, straightforward manner, that cannot be fully theoretically justified without the involvement of the explicit intention to learn principle, which was apparently present implicitly (by the fact of dealing with biologically secondary knowledge).

For instance, the worked example effect, which indicates that explicit instruction in solution steps is more efficient for learning domain-specific problem-solving schemas than learning by solving problems only (as it imposes less working memory load than search-based processes), was considered as the consequence of the borrowing and reorganizing principle and the randomness as genesis principle. A number of effects based on reducing working memory load in learning (such as split-attention effect, redundancy effect, or pre-training effect) were treated as the consequences of the narrow limits of change principle, while the expertise reversal effect according to which the effectiveness of different instructional techniques depends on levels of learner expertise in a specific domain was considered as the direct consequence of the information store principle and the environmental organizing and linking principle (Sweller et al., 2011).

The last, sixth principle distinguishing the intelligent natural information-processing systems from other, lower-level natural information-processing systems is responsible for controlled information processing and explicit learning as opposite to implicit cognitive mechanisms and is presumably present in all the above cognitive load effects since they are dealing with learning biologically secondary, explicit knowledge. It was suggested that this principle is also directly related to intelligence as an emergent characteristic of natural information processing systems which cannot be incorporated within the traditional five-principles model. Human intelligence is associated with explicit, intentional generation and learning of biologically secondary knowledge, which always requires the learner to be motivated and engaged in such activities. Together, these processes led to the emergence of human cumulative culture on a grand scale which defined the transition from biological to cultural evolution (see Kalyuga, 2025, for an overview of these processes).

Therefore, maintaining and further enhancing natural human intelligence is an important aspect of the explicit intention to learn principle. It becomes even more critical because of the following considerations. With the development of truly intelligent artificial information processing systems (strong AI), cultural evolution is believed to be entering its technological phase (e.g., Tegmark, 2017). In the future, it may remain the phase within cultural evolution, or it may transition into the next, post-cultural technological stage of evolution which could potentially make biological human intelligence redundant. If humans are unable to continuously improve their natural intelligence, they may eventually lose their evolutionary competitiveness to AI-based intelligent machines (Dick, 2003, 2008). Accordingly, the following subsection specifically focuses on this educational consequence of the explicit intention to learn principle–the need to strengthen human intelligence with the purpose of keeping it competitive and in control of artificial intelligence during the technological phase of cultural evolution.

### Educational Approaches to Strengthening Human Intelligence

In psychology, intelligence is generally treated as a broadly defined term that usually relates to the human capability to rapidly and effectively learn to handle completely novel situations across different domains (e.g., Gignac & Szodorai, 2024; Gottfredson, 1997; Legg & Hutter, 2007; Whiten & van Schaik, 2007). Because it relates to dealing with principally novel problems across domains, intelligence, or more precisely general intelligence, differs from expertise which assumes high levels of performance on already familiar types of tasks in a specific domain due to the acquisition of well-organized biologically secondary knowledge structures and extensive experience in the domain (Ericsson, 2006). Therefore, while the former, the ability to reason and problem solve in novel situations, is frequently defined as general fluid intelligence, the latter, as the accumulated organized secondary knowledge structures (e.g., facts, principles, procedures, and schemas) in the person’s long-term memory, is defined as general crystallized intelligence (Cattell, 1963).

A major part of human cognitive architecture that has been consistently associated with fluid general intelligence is working memory. It is directly involved in conscious, controlled problem-solving and reasoning. General fluid intelligence has been shown to be strongly related (most likely, causally) to characteristics of working memory such as its capacity (working memory span) (e.g., Conway et al., 2002; Hagemann et al., 2023; Kane & Engle, 2002), as well as attentional control and inhibitory processes (e.g., Burgoyne & Engle, 2020; Engle, 2002). The available prior secondary knowledge structures in long-term memory (as an attribute of crystallized intelligence) may affect reasoning and novel problem-solving in different ways, for example, by using analogies, applying domain-general principles, or through enhancing working memory processing capacity due to the chunking effect (i.e., encapsulating many elements of information into larger familiar units of knowledge). Vice versa, the above characteristics of fluid general intelligence (and related features of working memory) may affect the processes of acquisition of organized knowledge structures associated with crystallized intelligence. Therefore, boundaries between crystallized and fluid intelligence could not always be clear-cut, and they may well influence each other significantly.

Thus, it is possible to propose at least two major directions of strengthening learner general fluid intelligence. One is through enhancing crystallized intelligence: acquiring more extensive and better-organized knowledge structures in long-term memory could improve fluid intelligence in the above-mentioned ways: either directly (by offering analogies, general domain principles, etc. that could be applied to reasoning and novel problem solving) or indirectly (through enhancing the processing capacity of working memory due to the chunking effect). This direction is effectively related to the acquisition of domain-specific expertise-starting from the expertise in one or several very narrow areas (e.g., even within school subjects), then possibly extending it to a broader domain or domains. The means of enhancing the acquisition of expertise have been investigated in specialized theories of expertise (e.g., deliberate practice theory by Ericsson, 2006), as well as in general instructional theories (e.g., worked example effect, fading guidance effect, or expertise reversal effect in cognitive load theory, Sweller et al., 2011), or in combinations of both (e.g., Pachman et al., 2014).

Another way of enhancing general fluid intelligence is through directly improving components involved in this human capability, such as individual or group reasoning, inferencing, and means of novel problem-solving. This direction will be discussed in more detail here, as it has caused some contrasting opinions among researchers and educators. On the one hand, there are many university and business courses around (in both face-to-face and online modes) devoted to teaching general problem-solving strategies. Even though they are usually tied to specific domains (e.g., business or mathematics), still the methods offered are cross-domain and in most cases, based on variations of the means-ends strategy as a general problem-solving method (Newell & Simon, 1972) or general metacognitive approaches to solving novel problems (a classic example is the four-step problem-solving process by Polya, 1945).

On the other hand, a few instructional psychologists, particularly in cognitive load theory, believe that such general problem-solving skills belong to biologically primary abilities by nature and therefore, they do not need any explicit instruction. It is argued that, like with any other biologically primary skills, humans are predisposed to learn them fast, implicitly, just by being involved in corresponding types of activities, in this case, novel problem-solving in various domains (e.g., Sweller et al., 2011; Sweller, 2022). This opinion has been based on the randomness as genesis principle as one of the five original principles of operation of natural information processing systems applied to human cognitive architecture. As discussed in the previous section, in human cognitive architecture, when considered in the absence of influence of the explicit intention to learn principle, the randomness as genesis principle relates to the generation of new biologically primary knowledge through the random generation and testing procedure. In this case, the randomness as genesis principle indeed stipulates implicit operation in the form of evolved biologically primary abilities that do not need any explicit instruction to be learned: they are learned implicitly in the course of being involved in individual or group novel problem-solving activities (Geary, 2005; Sweller, 2022). For example, human (as well as other primates) babies successfully use such implicitly learned methods for solving novel everyday problems (e.g., when extracting a toy from a directly inaccessible location with the help of a long stick, etc.).

However, when applied in biologically secondary domains, this principle operates jointly with the explicit intention to learn principle (Figure 1), which stipulates conscious, explicit, effortful information processing in working memory. The primary, implicit abilities still could be helpful in this process, as conscious reflection on the implicit problem-solving methods could help in generating their more sophisticated, biologically secondary versions. But the act of consciously controlled reflection on primary knowledge is the process operating within the secondary domain, and like any biologically secondary information or ability needs to be learned explicitly. Therefore, general problem-solving heuristics such as means-ends analysis in the secondary domain require explicit learning, with all the instructional approaches to learning secondary knowledge applicable to the case, including cognitive load effects (e.g., the worked example effect, fading guidance effect, etc.). For this reason, it should not be contradictory to cognitive load theory to suggest worked examples in applying means-ends analyses to solving novel domain-specific problems as illustration of the technique. Such an approach could possibly be more cognitively efficient (i.e., generating less unnecessary cognitive/working memory load) for learners who are beginners in using the technique than just asking them to apply the approach in an unguided manner, by relying on their own reflection on their primary ability.

The same applies not only to means-ends analyses, but to all general problem-solving heuristics that have their origins in evolved, implicit primary intuitions (e.g., trial-and-error technique). Even more generally, it may also apply to human metacognitive skills, which, according to the traditional view of instructional consequences of human cognitive architecture in cognitive load theory, do not need much explicit instruction as belonging to biologically primary skills, like general problem-solving heuristics. Indeed, basic metacognitive abilities are biologically primary skills for many natural information processing systems governed by five original principles within the biologically primary domains. This includes not only humans but also great apes who are capable of metacognitive monitoring and controlling their decision-making processes (Carruthers & Williams, 2022; Tomasello, 2023). However, when such basic primary skills are consciously reflected upon by humans under the explicit intention to learn principle, they become part of the biologically secondary domain which requires explicit learning approaches, with all relevant cognitive load techniques applicable (e.g., using worked examples for initial instruction of novice learners, gradually replacing them with less-guided metacognitive prompts as learners become familiar with basic secondary metacognitive strategies).

Thus, even though basic general problem-solving heuristics and metacognitive skills applied by humans in consciously controlled ways using working memory under the explicit intention to learn principle may seem to be the same type of skills as evolved implicit biologically primary intuitions, they involve different types of mechanisms that make them explicitly learned heuristics. This opens the whole repertory of explicit instructional approaches (including evidence-based cognitive load theory techniques), as well as exploratory, problem-first, and self-regulated learning approaches (e.g., Bjork & Bjork, 2020; Bjork et al., 2013; Kapur, 2008) to be used for the improvement of these abilities which are critical for continuous enhancement of human intelligence.

## 4. Conclusions

The suggested architecture of human cognition as an intelligent natural information processing system (depicted in Figure 1) integrates implicit (primary) and controlled (secondary) processes within the same cognitive architecture involving a set of six principles, the first five of which would also apply to most other natural information processing systems. This architecture may allow us to naturally embed the role of motivation in the explicit, controlled processing of biologically secondary information, as well as intelligence (the ability to generate such information), and human culture (as a repository of secondary information that has accumulated over generations).

While instructional implications of the first five principles of natural information processing systems have been well researched within cognitive load theory for quite some time, the recently formulated sixth principle (explicit intention to learn) implies some additional educational consequences that need further research. This paper only briefly discussed enhancing human learners’ natural intelligence. This focus may boost learner abilities to effectively manage new content, adapt their existing implicit, primary skills to new contexts by intentionally reflecting on them, and possibly work collaboratively with others to boost “group intelligence”. It could therefore be important for future research to emphasize the intentionality of the learner in explicitly adapting known biologically primary approaches to new contexts.

Enhancing learner motivation to apply their intelligence to explicitly learn biologically secondary knowledge is another important educational implication of the explicit intention to learn principle, which requires separate discussion and further research (e.g., Would exploratory learning environments be effective for this and under which conditions in terms of necessary levels of instructional guidance, metacognitive support? What is the role of learner curiosity, mimicry, or play in learning in enhancing learner motivation?). These research directions could be grounded in both cognitive (e.g., expanding working memory, enhancing attentional distribution, chunking information, and strengthening inhibitory control to increase intelligence) and socio-constructivist (enhancing learners’ motivation and engagement) perspectives.

## Data Availability

Not applicable.
